# The Importance of the Mediastinal Triangle in Traumatic Lesions of the Aorta

**DOI:** 10.3390/medicina55060263

**Published:** 2019-06-10

**Authors:** Daniel Gulias-Soidan, Daniel Fraga-Manteiga, Víctor X Mosquera-Rodriguez, Milagros Marini-Diaz, Paula Lopez-Bargiela, Cristina González-Martín, Vanesa Balboa-Barreiro

**Affiliations:** 1 Department of Radiology, Complejo Hospitalario Universitario de A Coruña (CHUAC), As Xubias 84, 15006 A Coruña, Spain; daniel.gulias.soidan@sergas.es (D.G.-S.); daniel.fraga.manteiga@sergas.es (D.F.-M.); Milagros.marini.diaz@sergas.es (M.M.-D.); 2 Department of Cardiac Surgery, Complejo Hospitalario Universitario de A Coruña (CHUAC), As Xubias 84, 15006 A Coruña, Spain; Victor.x.mosquera.rodriguez@sergas.es; 3 Department of Orthopedic Surgery and Traumatology. Complejo Hospitalario Universitario de A Coruña (CHUAC), As Xubias 84, 15006 A Coruña, Spain; Paula.lopez.bargiela@sergas.es; 4 Clinical Epidemiology Research Group, Health Sciences Department, Faculty of Nursing and Podiatry. Universidade da Coruña (UDC), Campus de Esteiro, 15403 Ferrol, Spain; 5 Clinical Epidemiology and Biostatistics Unit, Biomedical Research Institute of A Coruña (INIBIC)-Universitary Hospital of A Coruña (CHUAC), SERGAS, University of A Coruña (UDC), As Xubias 84, 15006 A Coruña, Spain; vanesa.balboa.barreiro@sergas.es

**Keywords:** aorta, blunt trauma, thoracic trauma

## Abstract

*Background:* Trauma-induced aortic injuries continue to be an important factor in morbimortality in patients with blunt trauma. *Objectives:* To determine the characteristics of aortic lesions in patients with closed thoracic trauma and associated thoracic injuries. *Methods:* Multicenter cohort study conducted during the years 1994 to 2014 in the radiology service in the University Hospital Complex of A Coruña. Patients >15 years with closed thoracic trauma were included. Sociodemographic and clinical variables were studied in order to determine the lesion cause, location, and degree. *Results:* We analyzed 232 patients with a mean age of 46.9 ± 18.7 years, consisting of 81.4% males. The most frequent location was at the level of the isthmus (55.2%). The most frequent causes of injury were traffic accidents followed by falls. Patients with aortic injury had more esophageal, airway, and cardiopericardial lesions. More than 85% of the patients had lung parenchyma and/or chest wall injury, which was more prevalent among those who did not have an aortic lesion. *Conclusions:* Patients with trauma due to traffic accidents or being run over presented three times more risk of aortic injury than from other causes. Those with an aortic lesion also had a higher frequency of cardiopericardial, airway, and esophageal lesions.

## 1. Introduction

Trauma is the leading cause of death in those under 45 years of age in the United States and the European Union [1,2,3]. In Spain, traumatisms due to traffic accidents are the first cause of death in those under 34 years of age [4].

In polytraumatized patients, thoracic injuries are the third cause of injury with a mortality rate between 15,25%; if we add cardiac, tracheobronchial or esophageal lesions, the percentage may be higher [2].

The most frequent mechanism of injury associated with traumatic injuries of the aorta are car accidents, followed by being run over, motorcycle accidents, and falls [5].

There is a great controversy regarding what the true mechanism of injury in blunt trauma of the aorta; currently it is considered that the union of shearing forces with a rapid deceleration, hydrostatic forces, and the mechanism of the bone clamp help to promote this traumatism [6,7,8].

Due to the mechanism of production of the aortic lesions, all the thoracic structures are subjected to forces of pressure, traction or shearing that can cause lesions in other intrathoracic organs and in structures of the same thoracic wall. Among the possible injuries are lesions of the airways, pleural, pulmonary, esophageal or cardiac among others [9].

The importance of the mediastinal triangle is due to the important organs it contains that give it well-defined anatomicfunctional characteristics.

In recent years, there have been important changes in the diagnosis and treatment of aortic lesions and associated lesions, with computed tomography (CT) being the method of choice for diagnosis. The CT allows us to identify all the structures of the thorax and, with it, rapidly detect alterations associated with the aortic lesion, improving the clinicaltherapeutic management of these critical patients.

The main objective of this study was to describe the clinical characteristics of aortic lesions in patients with closed thoracic trauma and the associated thoracic injuries.

The secondary objective was to analyze the long-term survival in a multicentric study of closed thoracic trauma.

## 2. Materials and Methods

Multicenter cohort study was conducted during the years from 1994 to 2014 in the radiology service. We included patients older than 15 years, with closed thoracic trauma with an assessment of the severity of the injury greater than 15 according to the Injury Severity Score (ISS) [10] and who presented emergency CT in which the presence of an aortic lesion associated with trauma it could be objectified or ruled out. Patients who did not have CT at the time of the admission or those who had penetrating trauma were not included.

The group of cases consisted of all patients with associated aortic lesion (*n* = 58). Three controls per case were randomly selected from patients with closed thoracic trauma who did not have an aortic lesion (*n* = 174).

This sample size made it possible to detect as significant an OR ≥ 9.1 associated with the presence of esophageal injury, assuming a percentage of exposure between cases of 10%, a 95% safety, and a statistical power of 80%.

Variables were collected from each patient included in the study: sociodemographic (age, sex) and clinical (mechanism, location and degree of injury according to the classification of the Organ Injury Scale (OIS)) [11]. The OIS scale evaluates the severity of the injury individually, establishing a classification on a scale of 1 to 5–6 according to the injured organ where 1 represents the minor involvement and 6 the most serious injury.

### Statistical Analysis

A descriptive study of the variables collected in the study was carried out. The quantitative variables were expressed as mean ± sd, median, and range. The qualitative variables were expressed as frequency (*n*) and percentage with the estimation of the corresponding 95% confidence interval. The comparison of means between two groups was performed using the Student’s T test or Mann–Whitney test, as appropriate. The comparison of means between more than two groups was performed using the ANOVA test or the Kruskal–Wallis test as appropriate after checking normality using the Kolmogorov–Smirnov test.

The association between qualitative variables was estimated using the Chi-square statistical test or Fisher’s test as appropriate.

The study of possible impact factors in the response variable was carried out using univariate and multivariate logistic regression, adjusting for those variables that were significant in the univariate or clinically relevant analysis.

Estimates of survival were accomplished with Kaplan–Meier methods. Differences in probability of survival according to the type of injury were analyzed with the log-rank (Mantel–Cox) test.

## 3. Results

In the present study, 232 patients were analyzed, with a mean age of 46.9 ± 18.7 years and a predominance of males (81.4%). Table 1 shows the characteristics of the patients under study. Mean age of patients with aortic lesion (*n* = 58) was 43.66 ± 18.13 years. The most frequent injury mechanism in these patients was a car accident (50%), followed by a motorcycle accident (19%), and falls (15.5%). The most frequent localization in aortic lesions was at the level of the isthmus (55.2%). A total 29.3% of patients with an aortic lesion died. Patients without an aortic lesion presented a slightly higher mean age than patients with this lesion (47.94 ± 18.92 vs. 43.66 ± 18.13 years). Most frequent mechanism of injury was car accidents, as was observed in those with an aortic lesion. Regarding mortality, it was significantly lower in these patients, with respect to those with an aortic lesion (13.2% vs. 29.3%, *p* = 0.001).

All patients with aortic injury presented vascular injury of grade ≥III, and more than 80% of them also presented pulmonary parenchymal injury (29.2% grade ≥III) or chest wall injury (67.3% of grade ≥ III) (Table 2). Among patients without an aortic lesion, lesions of the pulmonary parenchyma (63.5% grade ≥III) and the chest wall (11.5% grade ≥III) predominated. All thoracic lesions, except for the diaphragm lesion, showed significant association with the presence of aortic lesion. Esophageal lesions, although less frequent, were more prevalent among patients who presented aortic lesion (5.3% vs. 0.6%, *p* = 0.047). The presence of airway and cardiopericardial lesions was determined as a risk factor. In this way, traumatisms with airway injury presented approximately twelve times greater risk of aortic injury (OR = 11.80), as having a cardiopericardial lesion also increases the risk of presenting aortic lesion (OR = 7), 48), compared to patients who did not suffer such injuries. Conversely, a lower risk of aortic injury was observed in patients with a chest wall injury (OR = 0.26) and lung parenchyma (OR = 0.20).

Taking into account the age, sex, and different types of lesions (Table 3), it was found that the variables with an independent effect to modify the probability of presenting injury in the aorta are the presence of chest wall lesion of the airways and cardiopericardial. Thoracic wall lesions are a protective factor against the probability of aortic injury (OR = 0.15), while having an airway (OR = 19.33) or cardiopericardial lesion (OR = 9.74) an increased risk of presenting aortic lesion, maintaining the effect observed in the univariate analysis (Table 2).

**Figure 1:**
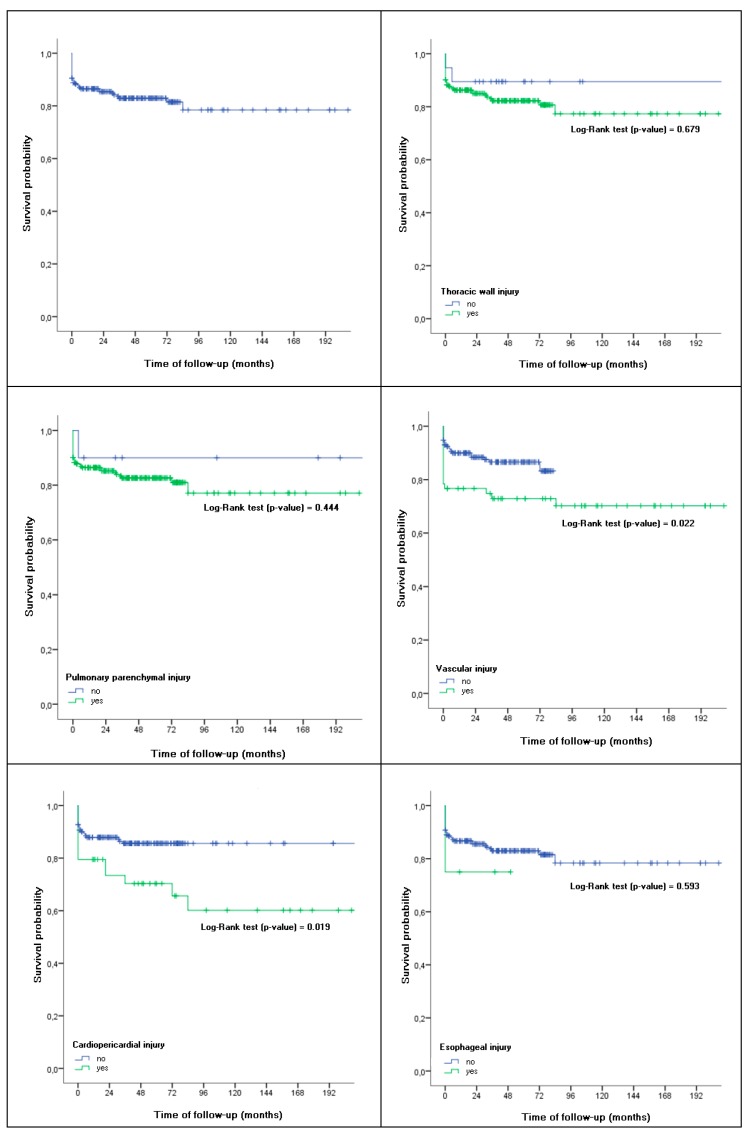
Survival in patients with thoracic trauma according to the type of injury.

The average survival time of these patients was 14.93 ± 0.59 years, resulting in an overall survival of 86.5%, 82.3%, and 78.4% per year, at three and ten years, respectively (Figure 1).

This survival pattern was similar in patients with a thoracic wall lesion or lung parenchyma lesion. In contrast, patients with a vascular injury or pericardial lesion showed a significantly lower survival rate than patients who did not present these lesions (*p* = 0.022 and *p* = 0.019, respectively). Survival in patients with vascular injury at one year, at three, and at ten years was 76.7%, 72.9%, and 70.2%, respectively, compared to 89.9%, 86.6%, and 83.2% of patients who did not have this lesion. The survival per year of the patients with a pericardial lesion was 79.5% vs. 87.9% in those who did not have it. This survival decreased in both groups (those with injury versus those who did not) to 70.3% vs. 85.6% at three years and to 60.2% vs. 85.6% at ten years.

## 4. Discussion

From the sample analyzed in our study, we found a majority of men, middle-aged, as in what was found in the literature, such as in the multicenter study by Alsac et al. [12].

With regard to the mechanisms of injury to the aorta, car accidents have been identified in several studies [13,14,15,16,17] as the most common mechanism responsible for a traumatic rupture of the aorta, data that agree with our results.

**Figure 2:**
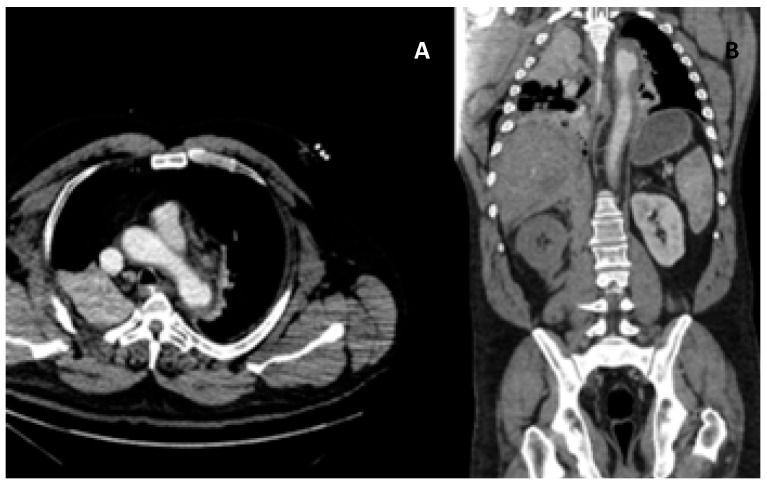
Collision trauma. Axial computed tomography (CT) (**A**) and coronal reconstruction (**B**) identify focal dissection and periaortic hematoma in the isthmus region.

Severe thoracic trauma can affect different structures such as the aorta, heart, vessels, etc. Several publications confirm that the aortic lesion occurs more frequently at the level of the isthmus [2,5,18] (Figure 2).

### 4.1. Injuries Associated with the Aorta

CT has been considered for years the method of choice for the diagnosis of traumatic injuries of the aorta and its associated injuries. The wide availability, speed, and accuracy of CT have meant an important improvement in the diagnosis and clinicaltherapeutic management of these patients.

The analysis and integral assessment of all the alterations that occur in blunt chest trauma is fundamental. CT can identify all the structures of the thorax and only through a systematized analysis of them will it possible to detect alterations associated with the traumatic injury of the aorta. The identification in CT of associated lesions can be a warning sign to maximize the search for other alterations.

Airway injuries are significantly associated with traumatic lesions of the aorta, as well as esophageal damage and lesions of the chest wall [19,20].

**Figure 3:**
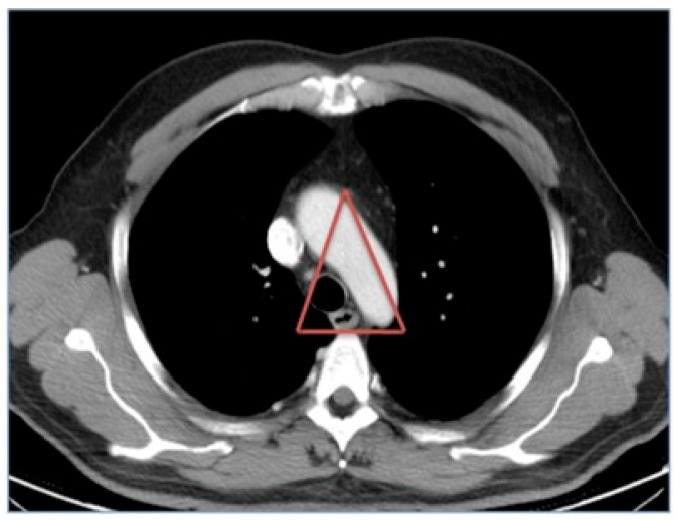
Mediastinal triangle in which the trachea, esophagus, and aorta are located.

Some authors have indicated that the low prevalence of esophageal and airway lesions in polytraumatized patients may be due to the frequent coexistence with vascular lesions, noting that this association may cause patients to die before reaching the hospital [18,19]. The esophagus and the airway are in the mediastinum, are of small transverse diameter, and are in a “protected” position, so it is difficult to injure them except in cases of high-energy impacts. In that case, the aorta may also be damaged, since the three structures are in a “neighborhood triangle” (Figure 3).

It can be assumed that the most susceptible injuries to the aorta are those of high energy, necessary to reach the mediastinum and the internal areas of the pulmonary parenchyma and that are sufficiently localized impacts so that this energy is not dispersed. Another possible explanation is that the underlying causes of most pulmonary contusions, combination of edema and interstitial and intra-alveolar hemorrhages secondary to gas expansion or acceleration and deceleration forces [21,22] are not enough to produce damage to the aorta.

### 4.2. Limitations of the Study

The findings of this study should be interpreted taking into account their possible limitations. To avoid a possible selection bias, all patients with closed thoracic trauma were included, excluding only those without CT performed in the emergency room on admission. Additionally, this it is a multicentric study that covers a very long period of time. The conclusions may not be extrapolated to other geographical areas, and the findings have varied throughout the period studied due to changes in the practice of care and the equipment used.

In relation to potential information biases, derived from the retrospective nature of the study, based on the review of medical records, a review was made by the research team of the CT images to contrast the information collected in the report. In cases of discrepancy, a third observer proceeded to review the images.

Finally, the purpose of the study was eminently descriptive. The impact of traumatism characteristics in the presence or absence of an aortic lesion was investigated by means of a multivariate analysis controlled only by sociodemographic data. The presence of other confounding factors that have not been contemplated in the present study cannot be ruled out.

## 5. Conclusions

The patients studied with thoracic trauma were mainly middle-aged males, victims of traffic accidents.

The most frequent lesion was the pulmonary parenchyma, followed by the thoracic wall.

The patients with traumatism due to a traffic accident or being run over presented three times more risk of injury of the aorta than those with trauma due to precipitation.

In those who presented an aortic lesion, a greater frequency of cardiopericardial, airway, and esophageal lesions was observed.

In the multivariate model, adjusting for age, sex, and the different types of lesions, it was found that airway or cardiopericardial lesions significantly increased the risk of presenting aortic lesions.

## Figures and Tables

**Table 1:** Characteristics of the patients under study according to the presence or absence of a lesion in the aorta.

	Injury AO			
	Yes (*n* = 58)	No (*n* = 174)		
	Mean ± SD	Mean ± SD	***p***	**OR (95% CI)**
**Age (years)**	43.66 ± 18.13	47.94 ± 18.92	0.145	0.99 (0.97–1.00)
	***n* (%)**	***n* (%)**		
**Sex**			0.639	
Woman	12 (20.7)	31 (17.9)		1.19 (0.56–2.52)
Man	46 (79.3)	142 (82.1)		1
**Mechanism of injury**			0.101	
Precipitation	9 (15.5)	59 (33.9)		1
Car accident	29 (50.0)	61 (35.1)		3.12 (1.36–7.14)
Motorcycle accident	11 (19.0)	31 (17.8)		2.33 (0.87–6.21)
Flattening	3 (5.2)	10 (5.7)		1.96 (0.45–8.54)
Run over	6 (10.3)	11 (6.3)		3.57 (1.06–12.17)
Other	0	2 (1.1)		
**Location of the Ao lesion**				
Upward	2 (3.4)			
Aortic camber	5 (8.6)			
Isthmus	32(55.2)			
Descending aorta	19 (32.8)			
**In-hospital exodus**			**0.008**	
No	41 (70.7)	151 (86.8)		1
Yes	17 (29.3)	23 (13.2)		2.72 (1.33–5.56)

Ao: Aorta.

**Table 2:** Characteristics of the lesions of the patients under study according to the presence or absence of a lesion in the aorta.

	Injury AO			
	Yes (*n* = 58)	No (*n* = 174)		
	***n* (%)**	***n* (%)**	***p***	**OR (95% CI)**
**Thoracic wall injury**			0.010	
No	10 (17.2)	9 (5.2)		1
Yes	48 (82.8)	165 (94.8)		0.26 (0.1–0.7)
Grade I–II	34 (70.8)	146 (88.5)		
Grade ≥III	14 (29.2)	19 (11.5)		
**Pulmonary parenchymal injury**			0.017	
No	6 (10.3)	4 (2.3)		1
Yes	52 (89.7)	170 (97.7)		0.20 (0.05–0.75)
Grade I–II	17 (32.7)	62 (36.5)		
Grade ≥III	35 (67.3)	108 (63.5)		
**Diaphragm injury**			0.167	
No	55 (94.8)	171(98.3)		1
Yes	3 (5.2)	3(1.7)		3.11 (0.61–15.85)
Grade I–II	1 (33.3)	1 (33.3)		
Grade ≥III	2 (66.7)	2 (66.7)		
**Vascular injury**			0.000	
No	0	172 (98.9)		
Yes	58 (100.0)	2 (1.1)		
Grade I–II	0 (0)	1 (50.0)		
Grade ≥III	58 (100)	1 (50.0)		
**Airway injury**			0.001	
No	51 (87.9)	172 (98.9)		1
Yes	7 (12.1)	2(1.1)		11.80 (2.37–58.59)
**Esophageal injury**			0.047	
No	54 (94.7)	173 (99.4)		1
Yes	3 (5.3)	1 (0.6)		9.61 (0.98–94.31)
Grade I–II	3 (100)	1 (100)		
**Cardiopericardial injury**			0.000	
No	34 (58.6)	159 (91.4)		1
Yes	24 (41.4)	15 (8.6)		7.48 (3.55–15.74)
Grade I–II	19 (79.2)	13 (86.7)		
Grade ≥III	5 (20.8)	2 (13.3)		

Ao: Aorta.

**Table 3:** Factors associated with the presence of aortic injury. Multivariate logistic regression.

	B	SE	*p*	OR (95% CI)
Age (years)	−0.011	0.010	0.259	0.98 (0.97–1.01)
Sex	0.020	0.468	0.965	1.02 (0.41–2.55)
Thoracic Wall injury	−1.864	0.556	0.001	0.15 (0.05–0.46)
Pulmonary Parenchymal injury	−1.230	0.818	0.132	0.29 (0.05–1.45)
Diaphragm injury	0.901	0.987	0.361	2.46 (0.35–17.03)
Airway injury	2.962	0.879	0.001	19.33 (3.45–108.28)
Esophageal injury	1.910	1.302	0.142	6.75 (0.52–86.65)
Heart-pericardium injury	2.276	0.425	0.000	9.74 (4.23–22.39)

B: coefficient; SE: Standard Error.

## References

[1] Mullinix A.J., Foley W.D. (2004). Multidetector Computed Tomography and Blunt Thoracoabdominal Trauma. J. Comput. Assist. Tomogr..

[2] Peters S., Nicolas V., Heyer C. (2010). Multidetector computed tomography-spectrum of blunt chest wall and lung injuries in polytraumatized patients. Clin. Radiol..

[3] Williams S.R., Perera P., Gharahbaghian L. (2014). The FAST and E-FAST in 2013: Trauma ultrasonography: Overview, practical techniques, controversies, and new frontiers. Crit. Care Clin..

[4] DGT (2009). Principales Cifras de la Siniestralidad Vial. http://www.dgt.es/Galerias/seguridad-vial/estadisticas-eindicadores/publicaciones/principales-cifras-siniestralidad/Lasprincipales-cifras-2017-Internet.pdf.

[5] Teixeira P.G., Inaba K., Barmparas G., Georgiou C., Toms C., Noguchi T.T., Rogers C., Sathyavagiswaran L., Demetriades D. (2011). Blunt thoracic aortic injuries: An autopsy study. J. Trauma.

[6] Crass J.R., Cohen A.M., Motta A.O., Tomashefski J.F., Wiesen E.J. (1990). A proposed new mechanism of traumatic aortic rupture: The osseous pinch. Radiology.

[7] Cohen A.M., Crass J.R., Thomas H.A., Fisher R.G., Jacobs D.G. (1992). CT evidence for the “osseous pinch” mechanism of traumatic aortic injury. AJR Am. J. Roentgenol..

[8] Creasy J.D., Chiles C., Routh W.D., Dyer R.B. (1997). Overview of traumatic injury of the thoracic aorta. Radiogr..

[9] Kaewlai R., Avery L.L., Asrani A.V., Novelline R.A. (2008). Multidetector CT of blunt thoracic trauma. Radiographics.

[10] Mikhail J.N., Harris Y.D., Sorensen V.J. (2003). Injury Severity Scoring: Influence of Trauma Surgeon Involvement on Accuracy. J. Trauma Nurs..

[11] Moore E.E., Cogbill T.H., Malangoni M.A., Jurkovich G.J., Shackford S.R., Champion H.R., McAninch J.W. (1995). Organ Injury Scaling. Surg. Clin. North Am..

[12] Alsac J.M., Boura B., Desgranges P., Fabiani J.N., Becquemin J.P., Leseche G., PARIS-VASC (2008). Immediate endovascular repair for acute traumatic injuries of the thoracic aorta: A multicenter analysis of 28 cases. J. Vasc. Surg..

[13] Parmley L.F., Mattingly T.W., Manion W.C., Jahnke E.J. (1958). Nonpenetrating Traumatic Injury of the Aorta. Circulation.

[14] Greendyke R.M. (1996). Traumatic rupture of aorta: Special reference to automobile accidents. JAMA.

[15] Ochsner M.G., Hoffman A.P., DiPasquale D., Cole F.J., Rozycki G.S., Webster D.W., Champion H.R. (1992). Associated aortic rupture-pelvic fracture: An alert for orthopedic and general surgeons. J. Trauma: Inj. Infect. Crit. Care.

[16] Hunt J.P., Baker C.C., Lentz C.W., Rutledge R.R., Oller D.W., Flowe K.M., Nayduch D.A., Smith C., Clancy T.V., Thomason M.H. (1996). Thoracic aortic injuries: Management and outcome of 144 patients. J. Trauma.

[17] Maggisano R., Nathens A., Alexandrova N.A., Cina C., Boulanger B., McKenzie R., Harrison A.W. (1995). Trauma rupture of the thoracic aorta: Should one always operate immediately?. Ann. Vasc. Surg..

[18] Young C.A., Menias C.O., Bhalla S., Prasad S.R. (2008). CT features of esophageal emergencies. Radiographics.

[19] Bryant A.S., Cerfolio R.J. (2007). Esophageal trauma. Thorac. Surg. Clin..

[20] Rojas C.A., Restrepo C.S. (2009). Mediastinal hematomas: Aortic injury and beyond. J. Comput. Assist Tomogr..

[21] Miller L.A. (2006). Chest Wall, Lung, and Pleural Space Trauma. Radiol. Clin. North Am..

[22] Kang E.Y., Müller N.L. (1996). CT in blunt chest trauma: Pulmonary, tracheobronchial, and diaphragmatic injuries. Semin. Ultrasound CT MR.

